# Non-prescription antibiotics dispensing by community pharmacies: implications for antimicrobial resistance

**DOI:** 10.1097/MS9.0000000000001388

**Published:** 2023-11-02

**Authors:** Furqan K. Hashmi, Sitaram Khadka, Gopal K. Yadav, Mash’hood Mahmood Khan, Saif Ullah Khan, Hamid Saeed, Mohammad Saleem, Santoshi Giri, Muhammad Fawad Rasool, Hussaam-ul-Haq Mansoor, Zineb Riboua

**Affiliations:** a Punjab University College of Pharmacy, University of the Punjab, Lahore, Punjab; b Shree Birendra Hospital, Nepalese Army Institute of Health Sciences, Kathmandu; c Narayani Sub-Regional Hospital, Birgunj; d Nepal Public Health Foundation, Kathmandu, Nepal; e Department of Pharmacy Practice, Faculty of Pharmacy, Bahauddin Zakariya University, Multan, Pakistan; f McCourt School of Public Policy, Georgetown University, Washington, District of Columbia, USA

**Keywords:** anti-bacterial agents, developing countries, drug resistance, microbial, Pakistan, pharmacy, public health

## Abstract

**Introduction:**

The non-prescription antibiotics dispensing (NPAD) from pharmacies is on the rise in low- and middle-income countries, which contributes to the emergence of antimicrobial resistance (AMR). This study was conducted with the objective to determine the community pharmacy personnel’s perspectives on NPAD and its implications for AMR.

**Methods:**

A questionnaire-based cross-sectional survey was conducted in Pakistan among 336 pharmacies. The data were analyzed using SPSS v21 and MedCalc for Windows v12.3.0. Modified Bloom’s cut-off point was utilized to categorize the participants’ overall knowledge, attitude, and practice. For univariable logistic regression analyses, odds ratio (OR) was calculated at 95% confidence interval (CI). For multivariable logistic regression analyses, adjusted OR was calculated at 95% CI. Spearman’s rank correlation coefficient test was used to assess the relationships among knowledge, attitude, and/or practice scores.

**Results:**

The majority of the respondents were staff pharmacists (45.5%). About four-fifths (78.9%) and half (50.9%) of the participants demonstrated moderate to good knowledge and practice, respectively. However, about only one-third (33.1%) had a moderate to good attitude. Staff pharmacists had higher odds of moderate to good knowledge (OR: 2.4, 95% CI: 1.2–4.7) and practice (OR: 2.3, 95% CI: 1.4–3.8). Total knowledge and practice (Spearman’s *ρ*: 0.280; *P* <0.001) and total attitude and practice (Spearman’s *ρ*: 0.299; *P* <0.001) scores were significantly correlated.

**Conclusion:**

The qualified pharmacists had satisfactory knowledge, attitude, and practices toward antibiotics. However, non-pharmacist staff lacked knowledge and had probable NPAD practice, which has a negative impact on public health. Regular refresher training, seminars, and strict enforcement of rules and regulations are essential.

## Introduction

Highlights

- Community pharmacy should be practiced under the proper leadership of qualified pharmacists.
- The educational intervention, regular refresher trainings, and exercises on the rational use of medicines, as well as the practice of professionalism for community pharmacy personnel, help ensure better practice.
- Already existing laws must be revitalized and strictly practiced to discourage the dispensing of non-prescription antibiotics.

The appearance of resistance to antibiotics is one of the greatest threats to public health, responsible for increased morbidity and mortality as well as increased healthcare costs^1,2^. The World Health Organization (WHO) has recognized the importance of resistance for a long time; therefore, in 2001, Global Strategy for Containment of Antimicrobial Resistance provided a framework of interventions to slow the emergence of resistance and reduce its spread^3^. Resistance to antimicrobials has been declared a global emergency; therefore, in May 2015, the 68th World Health Assembly endorsed a Global Action Plan (GAP) to combat antimicrobial resistance (AMR) and control further escalation in the trend^4^. The incidence of resistance to antibiotics is more prevalent in developing countries due to poor enforcement of laws and a lack of substantial surveillance systems^5^. This can be attributed to irrational use of antibiotics such as unnecessary, suboptimal (duration, frequency, indication, dose), and extensive use of broad-spectrum antibiotics^6,7^.

In low- and middle-income countries (LMICs), dispensing antibiotics without a prescription is a common trend^8^, as it is believed to be a convenient, time-saving, and cheap way of obtaining the drugs without a physician’s consultation^9^, consequently promoting the irrational practice which has long-term effects on patient’s health^10^.

Pakistan is the sixth most populous country and is categorized as LMIC with a Human Development Index (HDI) value of 0.560^11^. The healthcare system of Pakistan is under a constantly increasing burden of both communicable as well as non-communicable diseases, and a majority of healthcare spending is out of pocket^12,13^. In this context, the public finds it easy to get their medicines from local retail drug stores or pharmacies without having a legitimate prescription from a medical practitioner. Even antibiotics can be acquired as over-the-counter (OTC) medicines. A large number of broad-spectrum antibiotics dispensed through community pharmacies subsequently leading to antibiotic resistance has been documented in the literature^14^. The easy availability of antibiotics without prescription is assigned to poor affordability of the public to seek physician’s advice, previous experience with the same antibiotic, and poor enforcement of the prevailing laws such as Drug Sale Rules as well as Drug Regulatory Authority of Pakistan (DRAP) Act 2012^15,16^.

As far as the policy perspective in Pakistan is concerned, the Drug Act 1976/DRAP Act 2012 provides the legislative impetus for the sale of medicines only under the supervision of a registered pharmacist^16,17^. The Drug Sale Rules 2007^15^ are intended to regular the functioning of the pharmacies and ensure the provision of quality medicines to patients. These rules mandate the sale of drugs under the strict supervision of a qualified, registered, and licensed pharmacist^15^. Pakistan has endorsed the National Action Plan, 2017, for the containment of AMR, which includes seven key strategic priorities, operational plans, interventions, essential indicators of monitoring, and an evaluation framework^18^.

The present study aims to evaluate the knowledge, attitude, and practices of the community pharmacy staff about the non-prescription dispensing of antibiotics in the city of Lahore, Pakistan.

## Methods

### Ethics approval

The research has been performed in accordance with the Declaration of Helsinki. The ethics approval was obtained from the Institution. All the participants were made aware of their voluntary participation and confidentiality prior to the data collection. Informed consent to participate in the study was obtained from the participants.

### Study design and setting

A cross-sectional study was carried out in community pharmacies in Lahore, Pakistan, from January 2019 to September 2019, principally targeting antibiotics dispensing practice from the community pharmacy personnel without prescription.

It was conducted in accordance with the criteria of STROCSS 2021^19^.

### Study tool and grading method

A questionnaire was developed based on the in-depth literature review and the current prevalent practices in community pharmacies^20–23^. It comprised four sections: demographics of participants, knowledge, attitude, and practices of antibiotic dispensing. The knowledge section was based on the concept of the participants. The attitude section proclaims the thinking of the participant. The practice section presents what the respondents perform. Both attitude and practice sections contained statements on a five-point Likert scale. The statements that opposed the notion of knowledge, attitude, and practices were graded 5 points for strongly disagree and 1 point for strongly agree responses. Accordingly, the rest of the responses of disagree, unsure, and agree to 4, 3, and 2 points in decreasing order, respectively. Similarly, the statements that supported the notion of knowledge, attitude, and practices were graded 1 point for strongly disagree and 5 points for strongly agree responses. The rest of the responses were graded accordingly: 2, 3, and 4 for disagree, unsure, and agree responses, respectively.

The face and content validity of the questionnaire was carried out by three experts at the university. The reliability analysis yielded a Cronbach *α* value of 0.80.

Participants’ overall knowledge, behavior, and practice were categorized using Bloom’s cut-off point (as the score is moderate to good if ≥60% and poor if <60%)^24^. The maximum knowledge score is 6 and ≥60% of 6 (≥3.6) was considered a moderate to good knowledge score. The maximum attitude score is 40 and ≥60% of 40 (≥24) was considered moderate to a good attitude. Similarly, the maximum practice score is 40 and ≥60% of 40 (≥24) was considered moderate to good practice.

### Sample size

The online sample size calculator ‘Raosoft’ was used for calculating sample size at 50% response distribution and 5% margin of error at 95% confidence interval (CI). For 3500 pharmacies in Lahore^25^, the sample size was 347 pharmacies. It was the number of pharmacies selected rather than the staff. Pharmacies were selected by stratified random sampling based on the administrative towns of Lahore, each town representing one stratum. Simple random sampling was employed in each town to identify the individual pharmacies to be visited. Any person first contacted (staff) at the time of visit was considered a respondent of the study; it could be a pharmacist, technician, dispenser, assistant, or pharmacy manager. Due to the incomplete data and the problem in getting consent, the non-response rate was found to be 3%. Therefore, a total of 336 samples were considered for inclusion in the study (Table 1). The overall knowledge of the community pharmacy staff regarding antibiotics as powerful medicines to fight bacterial infection, prescription-only medicines (PoM) status, the optimal duration of treatment, and pharmacists’ advice to patients regarding the use of antimicrobials to strictly adhere to the treatment regimen were evaluated.

**Table 1:** Study samples from different administrative towns of Lahore.

Sr. No.	Towns of Lahore	Total pharmacies	Study sample
1.	Allama Iqbal Town	702	77
2.	Ravi Town	371	44
3.	Data Gunj Baksh Town	443	44
4.	Gulberg Town	375	39
5.	Cantonment	385	11
6.	Nishtar Town	180	22
7.	Aziz Bhatti Town	134	14
8.	Shalamar Town	288	29
9.	Wagha Town	174	19
10.	Samanabad Town	448	37
Total		3500	336

### Data analysis

Data were analyzed by IBM SPSS v21. MedCalc for Windows v 12.3.0 (MedCalc-Software, Mariakerke, Belgium) was also utilized for further analysis. The total and mean scores of knowledge, attitude, and practice were calculated and recorded into different categorical variables: the moderate to good and poor score groups for each knowledge, attitude, and practice. Socio-demographic characteristics of participants were presented as frequency and proportions. The *χ* ^2^ test was used to test for group differences. For binary logistic regression analyses, the odds ratio (OR) was calculated at 95% CI. Multivariable logistic regression was used to determine independent factors associated with moderate to good knowledge, attitude, and practice (all variables with *P* <0.20), and the adjusted odds ratio (AOR) was calculated at 95% CI. Box plots were drawn for the distribution of knowledge, attitude, and practice scores based on education level and areas of work. Spearman’s rank correlation coefficient test was used to assess the relationships among the knowledge, attitude, and/or practice scores.

## Results

A total of 400 questionnaires were distributed among the community pharmacies, and 336 were returned yielding a response rate of 84%. The majority of the study population consisted of male staff, 84.5% (*n*=284). Most of the respondents were staff pharmacists, 45.5% (*n*=153), whereas 35.7% (*n*=120) were managers. Only 18.8% (*n*=63) were technicians. The majority of the respondents had an experience of fewer than 2 years, 34.8% (*n*=117), while 24.1% (*n*=81) were of 6–10 years of experience and 20.8% (*n*=70) were of 3–5 years of experience. The majority of the respondents had a bachelor’s degree in pharmacy science (56.8%; *n*=191), followed by intermediates (25.9%; *n*=87), and then graduate/master (13.4%; *n*=45) (Table 2).

**Table 2:** Demographics of the study population (*N*=336).

Demographics	Frequency (*n*) Percentage (%)
Gender	
Male	284 (84.5)
Female	52 (15.5)
Marital status	
^a^Single	169 (50.3)
Married	167 (49.7)
Age (years)	
18–27	163 (48.5)
28–37	115 (34.2)
38–47	46 (13.7)
>47	12 (3.6)
Job status	
Manager	120 (35.7)
Staff pharmacist	153 (45.5)
Technician	63 (18.8)
Experience (years)	
<2	117 (34.8)
3–5	70 (20.8)
6–10	81 (24.1)
>10	68 (20.2)
Qualification	
Intermediate	87 (25.9)
Graduate/Master	45 (13.4)
Diploma of Pharmacy	13 (3.9)
B.Pharm/Pharm.D	191 (56.8)

a Single: unmarried or divorced.

### Knowledge assessment

The correct responses were reported in Supplementary Materials, Table S1 (http://links.lww.com/MS9/A289). The median knowledge score was 5 (25th to 75th percentiles: 4–6). About four-fifths of the participants (265, 78.9%) had moderate to good knowledge. Male participants had lower odds of good knowledge (OR: 0.2, 95% CI: 0.1–0.6) than females. Similarly, staff pharmacists had higher odds of moderate to good knowledge (OR: 2.4, 95% CI: 1.2–4.7) than managers. Participants with an educational level of bachelor’s and/or above in pharmacy had 4.1 times higher odds of good to moderate knowledge (OR: 4.1, 95% CI: 2.4–7.1) than those with intermediate or diploma in pharmacy (Table 3).

**Table 3:** Factors affecting the knowledge of the participants about the non-prescription dispensing of antibiotics (*N*=336).

	Knowledge		Univariable logistics regression		Multivariable logistics regression	
Characteristics	Poor (%)	Moderate to good (%)	OR (95% CI)	*P*	AOR (95% CI)	*P*
Age (years)				0.700^a^		
≤27	33 (20.2)	130 (79.8)	1 (Ref.)			
≥28	38 (22.0)	135 (78.0)	0.90 (0.53–1.52)	0.700		
Gender					0.003^a^	
Female	3 (5.8)	49 (94.2)	1 (Ref.)		1 (Ref.)	
Male	68 (23.9)	216 (76.1)	0.19 (0.06–0.64)	0.007	0.34 (0.10–1.22)	0.099
Marital status					0.647	
Single	34 (20.1)	135 (79.9)	1 (Ref.)			
Married	37 (22.2)	130 (77.8)	0.89 (0.52–1.49)	0.647		
Job role					<0.001^a^	
Manager	26 (21.7)	94 (78.3)	1 (Ref.)		1 (Ref.)	
Staff pharmacist	16 (10.5)	137 (89.5)	2.4 (1.2–4.7)	0.012	1.71 (0.75–3.88)	0.200
Technician	29 (46.0)	34 (54.0)	0.3 (0.2–0.6)	0.001	0.41 (0.20–0.82)	0.012
Experience (years)					0.138^a^	
≤5	34 (18.2)	153 (81.8)	1 (Ref.)		1 (Ref.)	
≥6	37 (24.8)	112 (75.2)	0.67 (0.39–1.14)	0.139	1.39 (0.74–2.59)	0.309
Educational qualification					<0.001^a^	
Intermediate or diploma in pharmacy	39 (39.0)	61 (61.0)	1 (Ref.)		1 (Ref.)	
Bachelor in pharmacy and above	32 (13.6)	204 (86.4)	4.07 (2.36–7.05)	<0.001	1.88 (0.91–3.87)	0.089

Adjusted for gender, job role, experience, and educational qualifications (*P*-value <0.20).

Age equal to and less than 27 years, female gender, being single, job role as a manager, experience equal and less than 5 years, and education level of intermediate or diploma were taken as controls.

a *P*-value from *χ*2 test.

In multivariable regression model, managers had higher odds of moderate to good knowledge (AOR: 2.4, 95% CI: 1.2–0.8, *P*-value: 0.012) than technicians.

### Attitude assessment

The response to each question on the attitude questionnaire was presented in Supplementary Materials, Table S2 (http://links.lww.com/MS9/A289). The median attitude score was 21 (25th to 75th percentiles: 18–25). About one-third of the participants (111, 33.1%) had a moderate to good attitude. Neither of the socio-demographic factors was the independent predictor of moderate to good attitudes (Table 4).

**Table 4:** Factors affecting the attitude of the participants about the non-prescription dispensing of antibiotics (*N*=336).

	Attitude		Univariable logistics regression		Multivariable logistics regression	
Characteristics	Poor (%)	Moderate to good (%)	OR (95% CI)	*P*	AOR (95% CI)	*P*
Age (years)					0.972^a^	
≤27	109 (66.9)	54 (33.1)	1 (Ref.)			
≥28	116 (67.1)	57 (32.9)	0.99 (0.63–1.56)	0.972		
Gender					0.559^a^	
Female	33 (63.5)	19 (36.5)	1 (Ref.)			
Male	192 (67.6)	92 (32.4)	0.83 (0.45–1.54)	0.559		
Marital status					0.969^a^	
Single	113 (66.9)	56 (33.1)	1 (Ref.)			
Married	112 (67.1)	55 (32.9)	0.99 (0.63–1.56)	0.969		
Job role					0.078^a^	
Manager	78 (65.0)	42 (35.0)	1 (Ref.)			1 (Ref.)
Staff pharmacist	111 (72.5)	42 (27.5)	0.70 (0.42–1.18)	0.181	0.70 (0.42–1.18)	0.181
Technician	36 (57.1)	27 (42.9)	1.39 (0.75–2.60)	0.298	1.39 (0.75–2.60)	0.298
Experience (years)					0.678^a^	
≤5	127 (67.9)	60 (32.1)	1 (Ref.)			
≥6	98 (65.8)	51 (34.2)	1.10 (0.69–1.74)	0.678		
Educational qualification					0.807^a^	
Intermediate or diploma in pharmacy	66 (66.0)	34 (34.0)	1 (Ref.)			
Bachelor in pharmacy and above	159 (67.4)	77 (32.6)	0.94 (0.57–1.54)	0.807		

Adjusted for job role (*P*-value <0.20).

Age equal to and less than 27 years, female gender, being single, job role as a manager, experience equal and less than 5 years, and education level of intermediate or diploma were taken as controls.

a *P*-value from *χ* ^2^ test.

### Practice assessment

The response to each question on the practice questionnaire was presented in Supplementary Materials, Table S3 (http://links.lww.com/MS9/A289). The median attitude score was 24 (25th to 75th percentiles: 20–27.8). About half of the participants (271, 50.9%) had moderate to good practice. Age more than 28 years (OR: 0.6, 95% CI: 0.4–0.9), male participants (OR: 0.3, 95% CI: 0.1–0.6), married participants (OR: 0.4, 95% CI: 0.3–0.6), and experience duration more than 6 years (OR: 0.4, 95% CI: 0.3–0.7) had lower odds of moderate to good practice. However, staff pharmacists (OR: 2.3, 95% CI: 1.4–3.8) as compared to managers and educational level bachelor and/or above in pharmacy (OR: 3.2, 95% CI: 2.0–5.3) had higher odds of moderate to good practice (Table 5).

**Table 5:** Factors affecting the practice of the participants regarding the non-prescription dispensing of the antibiotics (*N*=336).

	Practice		Univariable logistics regression		Multivariable logistics regression	
Characteristics	Poor (%)	Moderate to good (%)	OR (95% CI)	*P*	AOR (95% CI)	*P*
Age (years)					0.009^a^	
≤27	68 (41.7)	95 (58.3)	1 (Ref.)			1 (Ref.)
≥28	97 (56.1)	76 (43.9)	0.56 (0.36–0.86)	0.009	1.23 (0.61–2.47)	0.569
Gender					<0.001^a^	
Female	13 (25.0)	39 (75.0)	1 (Ref.)			1 (Ref.)
Male	152 (53.5)	132 (46.5)	0.29 (0.15–0.57)	<0.001	0.49 (0.23–1.02)	0.055
Marital status					<0.001^a^	
Single	65 (38.5)	104 (61.5)	1 (Ref.)			1 (Ref.)
Married	100 (59.9)	67 (40.1)	0.42 (0.27–0.65)	<0.001	0.51 (0.26–0.99)	0.047
Job role					<0.001^a^	
Manager	69 (57.5)	51 (42.5)	1 (Ref.)			1 (Ref.)
Staff pharmacist	56 (36.6)	97 (63.4)	2.34 (1.44–3.82)	0.001	1.11 (0.60–2.06)	0.733
Technician	40 (63.5)	23 (36.5)	0.78 (0.42–1.46)	0.433	1.00 (0.49–2.03)	0.997
Experience (years)					<0.01^a^	
≤5	75 (40.1)	112 (59.9)	1 (Ref.)			1 (Ref.)
≥6	90 (60.4)	59 (39.6)	0.44 (0.28–0.68)	<0.001	0.84 (0.46–1.54)	0.572
Educational qualification					<0.001^a^	
Intermediate or diploma in pharmacy	69 (69.0)	31 (31.0)	1 (Ref.)			1 (Ref.)
Bachelor in pharmacy and above	96 (40.7)	140 (59.3)	3.25 (1.98–5.34)	<0.001	2.28 (1.18–4.41)	0.015

Adjusted for age, gender, marital status, job role, experience, and educational qualifications (*P*-value <0.20).

Age equal to and less than 27 years, female gender, being single, job role as a manager, experience equal and less than 5 years, and education level of intermediate or diploma were taken as controls.

a *P*-value from *χ* ^2^ tests.

In multivariable regression model, married participants had statistically significant lower odds of moderate to good practice (AOR: 0.5, 95% CI: 0.3–1) than unmarried participants.

### Distribution of knowledge, attitude, and practice scores based on education qualifications

**Figure 1:**
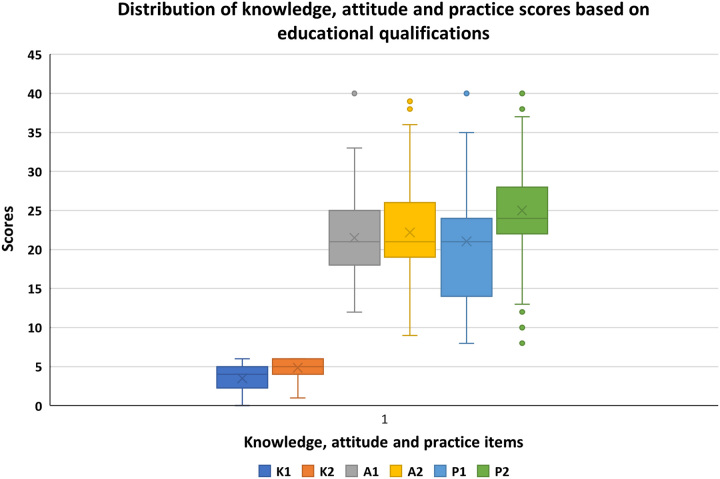
Box plots of the distribution of knowledge, attitude, and practice scores based on educational qualification (K1 = Knowledge ≤ Intermediate/Diploma in pharmacy and K2 = Knowledge ≥ Bachelor; A1 = Attitude ≤ Intermediate/Diploma in pharmacy and A2 = Attitude ≥ Bachelor; and P1 = Practice ≤ Intermediate/Diploma in pharmacy and P2 = Practice ≥ Bachelor).

Those participants with bachelor’s and above educational levels had higher median knowledge and practice scores as compared to those with lower educational levels (Intermediate/Diploma in Pharmacy). However, the median attitude scores were comparable across the two groups based on educational level (Fig. 1).

### Distribution of knowledge, attitude, and practice scores based on experience duration

**Figure 2:**
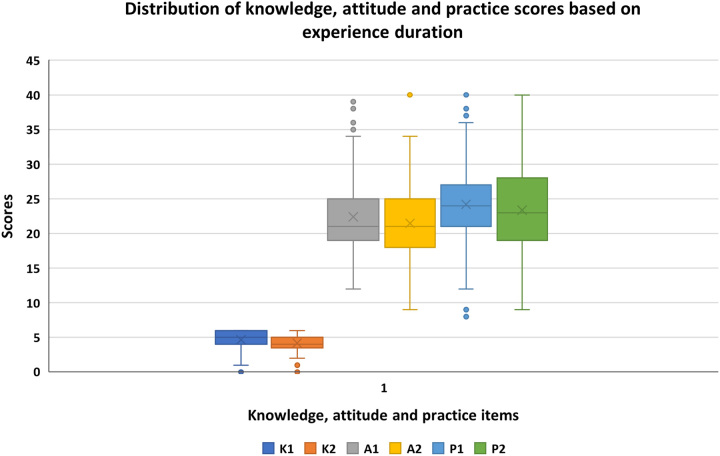
Box plots of the distribution of knowledge, attitude, and practice scores based on experience duration (K1 = Knowledge ≤5 years and K2 = Knowledge ≥6 years; A1 = Attitude ≤5 years and A2 = Attitude ≥6 years; and P1 = Practice ≤5 years and P2 = Practice ≥6 years).

Those participants with 6 or more years of experience had lower median knowledge and practice scores as compared to those with 5 or fewer years of experience. However, the median attitude scores were comparable across the two groups based on experience duration (Fig. 2).

### Correlation between the scores

There was no correlation between total knowledge and attitude scores (Spearman’s *ρ*: −0.052; *P*-value: 0.345). However, there was a significant correlation between total knowledge and practice scores (Spearman’s *ρ*: 0.280; *P*-value: <0.001), and total attitude and practice scores (Spearman’s *ρ*: 0.299; *P*-value: <0.001).

## Discussion

The disruptive implications of antibiotic resistance are already observed across the globe. With the emergence of resistant strains of bacteria, non-prescription antibiotic dispensing (NPAD) is also considered a relevant factor to deal with. It contributes substantially to the prevalence of AMR globally, both in the community and hospital settings^26^. Various studies all over the world demonstrated significant NPAD practice^21,27–31^. The different studies reported that a majority of pharmacies encountered visitors asking for antibiotics themselves without a prescription and about half of them dispensed antibiotics without any due requirements^32,33^. A similar trend was observed in this study. It is a common practice in the study setting and the rest of the country that people can get antibiotics sans legitimate prescriptions from a registered medical practitioner from any medical outlet whether they have a licensed pharmacist or not. A simulated client study from Pakistan reported that in a majority of the cases, the antibiotics are reported on the advice of the medicine store staff while less frequently dispensed from the pharmacies that are pharmacist-supervised^34^. The results of this study focus on the attributes that lead to NPAD at community pharmacies.

Out of 336 pharmacies visited, a majority had pharmacy graduates (56.8%) holding either a doctor of pharmacy (Pharm.D) or a bachelor of pharmacy (B.Pharm) degree working either as a manager or staff pharmacist while 43.2% of pharmacies visited had technicians or other pharmacy staff. The majority of the pharmacy staff, including pharmacists, had less than 2 years of experience. The unavailability of licensed pharmacists in the pharmacy might contribute to the NPAD resulting in the injudicious use of antibiotics that may lead to an increase in antibiotic resistance.

In Pakistan, the medicine stores are, to some extent, run by non-technical personnel and pharmacy technicians. The government has given a grace period of 10 years to upgrade their medicine stores to pharmacies. In addition to that, these sale rules have categorized the medicines into different schedules namely A, B, C, G, and F. The medicines included in Schedule-G are those which are to be sold under the strict supervision of a qualified pharmacist, and this schedule includes antibiotics, biologics, blood products, and vaccines. A non-technical person or a technician cannot sell these medicines as per the rules^35^.

Despite the drug-related rules and regulations like Drug Act 1976, DRAP Act 2012, and Drug Sale Rules 2007 being in place, the sales of antibiotics are poorly regulated because of poor enforcement by the concerned authority. This consequently leads to dispensing antibiotics without the prescription of a registered medical practitioner.

Knowledge of the participants, and staff pharmacists, on antibiotic use and dispensing was satisfactory. However, a fair portion of managers and technicians depicted poor knowledge, which may add to the unnecessary and over-dispensing of antibiotics from such personnel^36^. Our study revealed that 75% of the pharmacy staff were well aware of the PoM status of antibiotics. The majority of the respondents (75.9%) in this study agreed that antibiotic resistance is a serious public health problem. However, dispensers usually dispense antibiotics as OTC even though having known the PoM status. It is, therefore, necessary that the PoM status of antibiotics should be enforced especially concerning the sales from community pharmacies, to create an enabling environment for healthy behavior. A coordinated approach amongst stakeholders, including healthcare workers, patients, and policy-makers, on the issue of antibiotics sales and use is required^37^. Therefore, a multipronged approach with intra- as well as inter-professional coordination (IPC) can ensure compliance with the National Action Plan on AMR.

The moderate to good attitude demonstrated by only one-third (33.1%) of the respondents indicates the chances of misuse of antibiotics by pharmacy staff. It demands a need for refresher training and workshops on the rational use of drugs and the practice of professionalism among the pharmacy staff. A difference of opinion is detected among respondents on whether to use antibiotics for minor ailments or not. A good portion of participants stated that antibiotics dispensing without prescription renders ease to patients who visit pharmacies and for those who find it difficult to access healthcare facilities either due to lack of time or affordability^38^. Different studies across the globe evince a good perception of pharmacy staff regarding the use of antibiotics, but still, they do dispense without a physician’s order without any reluctance^39^. Our findings are found coherent with these facts. Results showed a prominent agreement of proper guidance of patients regarding the disease state of the patient, drug interactions, and emphasis on the complete course of antibiotics treatment, which is contradictory to the findings of Asian countries as retrieved by the public opinion about practices of pharmacy staff, especially the pharmacists^40^. About one-third of the respondents stated to dispense antibiotics even for self-limiting and viral infections, which is irrational. On average, more than half of the respondents agreed to provide antibiotics for infectious diseases if the patient gives a direct visit to their pharmacy as soon as the symptoms appear, where only pharmaceutical treatment of symptoms can bring relief. The view of respondents in our study coincided with the fact that there is a need for proper laboratory tests for pathogen identification and sensitivity analysis for initiating antibiotics, but their practice of NPAD does not match this. Such practice accounts for the irrational use of antibiotics in the community as indicated by National Institute for Health & Care Excellence (NICE) guidelines^41^. Moreover, guidelines suggest using diagnostic tools for assessing the severity of the disease, which will eventually reduce the overuse of antibiotics and avoid the emergence of resistance^42–44^.

Interventions are always a choice for improvement^45^. Good pharmacy practice (GPP) is a landmark standard for achieving rational, cost-effective, and responsible use of medicines to counter the abuse of antibiotics and halt AMR. In Pakistan, regarding AMR, the Ministry of National Health Services Regulations & Coordination Government of Pakistan (MNHSRC) devised a National Action Plan in the year 2017, in which weak curricula of professional education and lack of AMR awareness among professionals are pinpointed as weaknesses of the system^18^. Our findings are in line with the results presented in a study conducted among the physicians of Lahore, which iterates awareness about antibiotics to all the stakeholders of the healthcare profession^46^. The study demonstrated that low-dose and extensive use of broad-spectrum antimicrobials due to ease of access from pharmacies is responsible for high rates of AMR in Pakistan^46^. Another study conducted by Saleem *et al*.^14^ in Lahore demonstrated that co-amoxiclav, levofloxacin, ciprofloxacin, and third-generation cephalosporins were among the most widely consumed antimicrobials from private healthcare institutions and pharmacies. If the use of such antimicrobials goes unchecked, it results in more resistant strains of microorganisms such as the outbreak of extensively drug-resistant (XDR) Typhoid in Hyderabad, Pakistan, in 2016^47^.

According to a broad brush estimate of the global economic cost of antimicrobial drug resistance by 2050, the continually escalated resistance by 2050 will contribute to the death of 10 million people per year. There will be a reduction of 2–3.5% in Gross Domestic Product (GDP), which would cost the world up to 100 trillion United States Dollars^48^. In this context, the role of health authorities is very important; the Government of Pakistan should prioritize the issue of escalating AMR and formulate its containment strategies; however, there have not been sufficient resources allocated so far for this^49^. Efforts should be made to educate healthcare professionals who deal with drugs, such as pharmacists and physicians, regarding the appropriate use of antibiotics to reduce the likelihood of AMR^50^. Moreover, pharmacists should be present in the pharmacies and should educate the visitors about self-care and AMR as well. The patients or the public also need to be educated about the use of antibiotics upon prescription by a registered medical practitioner^51^.

### Strengths and limitations

This study signifies the importance of pharmacists and other staff involved in dispensing antibiotics at community pharmacies. The study setting is the second-largest city in LMIC Pakistan. The city is the center of culture, business, and education and has a structured and well-defined network of pharmacies with the highest number of pharmacists. Therefore, the generalizability of the study may not be ensured in rural areas and other countries with different health setups. Also, the respondents tend to provide socially desirable responses when they know their practices are being evaluated and may also not mention true practices, which is an inherent bias in the questionnaire-based study.

## Conclusion

The overall knowledge and practices of pharmacists working in pharmacies regarding dispensing of antibiotics were satisfactory, while staff other than pharmacists were mostly responsible for non-prescription dispensing of antibiotics in various conditions. Therefore, regular refresher training, tabletop exercises, and workshops on the rational use of medicines and the practice of professionalism must be conducted for the pharmacy personnel. Educational interventions are required for the public as well. The presence of a pharmacist should be made mandatory at all pharmacies, and strict enforcement of the DRAP Act 2012 and Drug Sale Rules 2007 is required for the rational use of medicines.

## Ethical approval

The research has been performed in accordance with the Declaration of Helsinki. The ethics approval was granted by the Humans Ethics Committee (HEC) University College of Pharmacy, University of the Punjab, Lahore (No: HEC/UCP/2045-19).

## Consent

All the participants were made aware of their voluntary participation and confidentiality prior to the data collection. Written informed consent was obtained from the participants for publication and any accompanying images. A copy of the written consent is available for review by the Editor-in-Chief of this journal on request.

## Sources of funding

This article did not receive any kind of grants.

## Author contribution

F.K.H.: conceptualization, methodology, investigation, supervision, and writing – original draft; S.K.: conceptualization, methodology, formal analysis, and writing – original draft; G.K.Y.: data curation, formal analysis, methodology, and writing – original draft; M.M.K. and S.U.K.: data curation, investigation, and writing – original draft; H.S. and M.S.: investigation, visualization, and writing – review and editing; S.G.: formal analysis, methodology, and writing – review and editing; M.F.R. and H.H.M.: investigation, supervision, and writing – review and editing; Z.R.: methodology, formal analysis, and writing – review and editing.

## Conflicts of interest disclosure

The author declare no conflicts of interest.

## Research registration unique identifying number (UIN)

1. Name of the registry: Research Registry.
2. Unique identifying number or registration ID: Researchregistry9005.
3. Hyperlink to specific registration (must be publicly accessible and will be checked): https://www.researchregistry.com/browse-the-registry#home/registrationdetails/645a6e66d054c200294aa708/.

## Guarantor

Sitaram Khadka, E-mail: sitaram.khadka@naihs.edu.np; Tel.: +977-9851077589. Address: Shree Birendra Hospital, Nepalese Army Institute of Health Sciences, Kathmandu, Nepal.

## Data availability statement

The data are available through the corresponding author on reasonable request.

## Provenance and peer review

All authors agree to the publication of the peer-review file for transparency.
